# A qualitative assessment of key considerations for drug checking service implementation

**DOI:** 10.1186/s12954-023-00882-y

**Published:** 2023-10-17

**Authors:** Chloe Grace Rose, A. Simon Pickard, Victoria Kulbokas, Stacey Hoferka, Kaitlyn Friedman, Jennifer Epstein, Todd A. Lee

**Affiliations:** 1 Department of Pharmacy Systems, Outcomes, and Policy, University of Illinois Chicago, 833 S Wood St, Chicago, IL 60612 USA; 2 Office of Policy, Planning, and Statistics, Illinois Department of Public Health, 122. S. Michigan Ave, 7th Floor, Chicago, IL 60603 USA

**Keywords:** Drug checking, Drug checking service, Harm reduction, Opioids

## Abstract

**Background:**

With many drug-related deaths driven by potent synthetic opioids tainting the illicit drug supply, drug checking services are becoming a key harm reduction strategy. Many drug checking technologies are available, ranging from fentanyl test strips to mass spectrometry. This study aimed to identify key considerations when implementing drug checking technologies and services to support harm reduction initiatives.

**Methods:**

Key informant interviews were conducted with harm reduction stakeholders throughout Illinois. Participants included members of existing drug checking services and recovery centers. Interviews were recorded, transcribed, and coded by two researchers using the framework method. Findings were contextualized according to micro (client)-, meso (organization)-, and macro (policy)-level themes.

**Results:**

Seven interviews were conducted with ten participants. Fourier transform infrared spectroscopy was consistently identified as a technology of choice given its accuracy, range of substance detection, portability, and usability. Recommendations included the use of confirmatory testing, which can help address the limitations of technologies and provide a mechanism to train technicians. Locations of drug checking services should maximize public health outreach and leverage existing harm reduction agencies and staff with lived experience, who are critical to developing trust and rapport with clients. Criminalization and loss of privacy were major concerns for clients using drug checking services. Additional issues included the need to raise awareness of the legitimacy of services through public support from governing bodies, and funding to ensure the sustainability of drug checking services.

**Conclusions:**

This research facilitated the identification of issues and recommendations from stakeholders around key considerations for the adoption of drug checking technologies, which not only included the cost and technical specifications of instrumentation, but also broader issues such as accessibility, privacy, and well-trained personnel trusted by clients of the service. Successful implementation of drug checking services requires knowledge of local needs and capacity and an in-depth understanding of the target population.

## Background

The opioid epidemic continues to be a major problem in the USA, with more than 107,000 deaths from drug-related overdose occurring in 2021, and more than 932,000 since 1999 [1, 2]. The nature of the epidemic has expanded from deaths caused by prescription opioids to mainly illicit opioids, such as fentanyl and its analogs. Between 2020 and 2021, overdose deaths involving synthetic opioids (excluding methadone) increased by 22%, while heroin-related deaths declined by 32%, highlighting an ongoing and dangerous trend in the illicit drug market and the opioid epidemic [3]. Fentanyl and other toxic adulterants in the illicit drug supply are leading to accidental overdoses and deaths [4]. People engaging in substance use may be exposed to drugs they did not intend to take, as illustrated in a recent article that described three separate incidents of New Yorkers dying from a drug-related overdose after ordering cocaine from a drug delivery service that was tainted with fentanyl. [5]

Given the pervasiveness of the opioid epidemic, there is an increasing need for harm reduction services to play a role in mitigating drug-related harms. Drug checking services have been utilized as a harm reduction strategy by providing information about the makeup of drug samples before or after use [6]. Specifically, drug checking may help people identify contaminants, assess dosage, and make safer choices. Additionally, harm reduction organizations may use drug checking services to engage their communities and collect data about current drug trends in the market that may impact local users [7, 8]. Monitoring trends within the drug market may allow for harm reduction and public health organizations to share drug alerts or warnings about the current illicit drug supply. [9, 10]

Currently, there are several technologies available to use for drug checking [11]. Fentanyl test strips are a simple and inexpensive drug checking technology to test for fentanyl and may be used with minimal training [12]. Test strips are available for other drugs and chemical reagents with similar characteristics and testing techniques to fentanyl test strips and likewise do not produce quantitative results. Testing with more powerful drug checking technologies, such as mass spectrometry (MS) and Fourier transform infrared (FTIR) spectroscopy instruments, could provide comprehensive information about what is contained in drug samples. [11]

A systematic review of the drug checking literature found that > 70% of studies were from Europe, while only 10% were from the USA [13]. While less frequent, studies published on US and Canadian drug checking services have highlighted barriers to drug checking for clients and staff members [8, 14–17]. This includes factors such as the location and hours of operation of a drug checking service [8, 14, 16, 17], the service modality (mobile vs. fixed locations) [8, 14, 15], destruction of drug samples during testing [8, 14], type of results provided (qualitative vs. quantitative) [8, 14, 17], fear of criminalization [8, 15], and concerns over privacy and anonymity [8]. The existing literature suggests that local context is critical to successful implementation. Consultation with stakeholders, particularly existing organizations who have established trusted relationships with the community, may inform how a drug checking service is implemented by anticipating potential barriers encountered. For example, the visibility of the location of a drug checking service in a community can negatively impact utilization of the service [18]. In addition, these factors may be further complicated by the context or environment of the drug checking service, such as the population it serves or the presiding legislation. There is a critical need to determine the value, benefits, and challenges of drug checking technologies and services given their importance as a harm reduction strategy amidst the opioid epidemic and the limited resources available. The aim of this study was to identify and understand key considerations for the implementation of drug checking services, from the perspective of USA-based stakeholders.

## Methods

### Study overview

Our qualitative study was centered on the content analysis of semi-structured interviews conducted with various harm reduction stakeholders across Illinois. This evaluation was conducted following a multi-year pilot study of a newly implemented drug checking service in an established urban harm reduction agency. The updated Consolidated Framework for Implementation Research (CFIR) [19] was used as a guiding framework for selection of initial topics in the interviews. CFIR was selected given our interest in learning about perceptions related to implementation, and previous application in qualitative research on drug checking [15]. A semi-structured interview guide was developed with four overarching domains: organization-specific information, drug checking technologies, the value of drug checking, and barriers and facilitators to drug checking. The development of the interview guide was an iterative process, informed by a scoping review of the literature [20] and comments from subject matter experts: a Senior Epidemiologist and Senior Health Scientist of the Centers for Disease Control and Prevention’s (CDC) Division of Overdose Prevention. All research activities were approved by the Institutional Review Board at the University of Illinois Chicago before the start of the study (Protocol #STUDY2022-1222).

### Population

Potential interview participants identified from harm reduction partnerships established by the Illinois Department of Public Health (IDPH) were invited to attend an optional presentation on drug checking from a local drug checking service, where information on this study was provided. A convenience sampling technique was utilized where participants were self-selected and asked to contact the research team if they were interested in joining the study. Eligibility criteria included representatives from organizations that provided harm reduction services and had experience providing or organizing services for their community. Services included, but were not limited to: drug checking, naloxone distribution, medication-assisted therapy (MAT), overdose education, distribution of sterile supplies, and counseling. The current use of drug checking technologies was not required for study participation as future interest and the importance of providing such a service were to be assessed. Study participants gave informed consent prior to participation.

### Data collection

The semi-structured interviews were conducted by three members of the research team that either had past experience in conducting key informant interviews or were trained on the process in preparation for conducting this study. Interviews lasted approximately one hour in duration and were completed virtually January 2023 through February 2023. Interviews were completed until the responses from participants repeatedly aligned with other participant responses and themes identified previously through the scoping review [20]. Interviews were audio-recorded, and transcripts were generated using virtual meeting software (Webex) [21]. All transcripts were manually reviewed and edited against audio recordings.

### Data analysis

Transcripts were analyzed using NVivo (2020) by study team members trained in qualitative methods (CGR and VK) [22]. We utilized inductive and deductive methods of the framework method to organize codes into a working analytical framework, allowing for data comparison across and within individual transcripts [23, 24]. Authors met frequently during the interview period to discuss identified themes and sub-themes, which were used to formulate a qualitative codebook. CGR and VK independently coded all transcripts and met to resolve any coding discrepancies. The findings were contextualized according micro-, meso-, and macro-level themes. Micro-level themes encompass considerations at the level of drug checking service clients, meso-level themes at the level of the drug checking service, and macro-level themes at the greater inter-organizational or state/policy level.

## Results

Seven interviews were conducted with 10 participants between January and February 2023. Participants included staff members of SSPs actively offering drug checking services (7), recovery or harm reduction centers providing limited, or no drug testing supplies (2), and a state-level organization experienced in emergency response and public safety, including response to the opioid epidemic (1). The participants engaged in this study held a variety of roles. The organizations had variable staff sizes, resources, and infrastructure for drug checking. All participating SSP members provided drug checking services to varying extents, with only one service utilizing quantitative technologies. However, all participating SSP members were well informed on the available quantitative technologies and were actively seeking or had recently obtained funding for quantitative technology acquisition. Results obtained from the interviews are contextualized into three major subheadings below, which correspond to micro-, meso-, and macro-level themes. An overview of major themes is provided in Table 1.

**Table 1:** Overview of major themes for implementation of drug checking services

Level	Identified Themes	Key Points
Micro (Considerations at the client level)	Accessibility	Mobile services are desirable to increase accessibility
	Privacy	Maintaining client privacy and anonymity are fundamental to drug checking service success
	Lived Experience	Lived experience was emphasized as a highly desirable characteristic of drug checking service staff to promote trust
Meso (Consideration at the drug checking service level)	Technologies	FTIR^a^ was the most suggested technology for drug checking. Participants emphasized quantitative capabilities, the wide breadth of substances that can be detected, portability, and lower cost compared to mass spectrometry
	Confirmatory Testing	Confirmatory testing is necessary to overcome technical limitations to drug checking technologies and to aid in validation of technician training
	Staff: Experience and Training	Standardization of technician training was stressed by participants
	Location	Candidates for offering drug checking services are preferably existing harm reduction agencies trusted by potential clients in locations that maximize the potential public health impact
	Funding	Integration of drug checking with other services is crucial to provide plausible deniability for clients due to stigma
	Integration of Services	Self-evaluation is important to ensure client-centered services
	Program Evaluation	Lack of funding remains a major barrier to drug checking services
Macro (Considerations at the inter-organizational, state or policy level)	Data Sharing	Great interest was expressed in data sharing between drug checking services to better inform clients of drug trends
	Partnerships	Partnerships were suggested to facilitate confirmatory testing, and data sharing
	Legality	Legality of samples for testing remains a major barrier for clients and drug checking service staff
	Legitimacy	Recognition of the legitimacy of harm reduction services is crucial to increase utilization and promote public health
	Advisory Panel	Forming an advisory panel for drug checking implementation may be a beneficial resource, but caution should be expressed to ensure no conflicts of interest

^a^Fourier transform infrared spectroscopy

### Micro-level themes

#### Accessibility

Addressing barriers to drug checking service accessibility was a common point of emphasis by participants. Mobility was highlighted as a key facilitator, as participants felt it would be unreasonable for clients to travel long distances to receive drug checking services. One participant noted how mobile services should be implemented with consideration to promote inclusivity, stating that “we have a lot of folks that live with disabilities and having a mobile van that doesn't have a lift could be a barrier”—Participant 2. Similarly, having broader hours of operation was described as important, with participants recognizing that drug use does not only occur during regular business hours.

The mobility of drug checking services can help to serve a secondary purpose for clients who fear surveillance and stigma associated with utilization of services at a fixed site. However, participants recognized that stigma is not specific to any single service modality and can occur at both mobile and fixed-site locations. The creation of plausible deniability for clients, through the integration of drug checking with other harm reduction services, was recommended for both mobile and fixed drug checking service locations to help combat stigma (described further in sections on Integration of Services and Privacy).

“There's a lot of surveillance. Whether they want to confirm or deny, and I think that requiring a brick and mortar would deter participants from accessing those services.”—Participant #1

#### Privacy

Maintaining client privacy was seen as fundamental by participants, who described the stigma clients face when accessing drug checking services. Ensuring private and anonymous access to drug checking has important implications for clients and the service modality used. Existing stigma, fear of being labeled as a drug user, and fear of surveillance and criminalization are major barriers for clients. To address these barriers, drug checking services must be discreet and offer clients plausible deniability. Protecting client anonymity also impacts how data is collected and shared. Participants described the importance of data sharing but emphasized that identifiable data should not be collected on clients (discussed in the section on Data Sharing).

“Prioritizing the anonymity of our participants, 100%. That is not negotiable.”—Participant #1.

#### Lived experience and trust

Lived experience was frequently identified by participants as a crucial characteristic of staff members. Participants described how understanding and relating to client experiences help form connections and build trust. Moreover, participants expressed how a lack of lived experience may negatively affect the drug checking service.

“Having some level of lived experience is pretty beneficial. Because otherwise you risk having them feel “othered” and even if you don't have that open stigma, sometimes it's just you lack that understanding.”—Participant #9

### Meso-level themes

#### Technologies

Participants expressed the need for more advanced technologies to better serve their communities. Specifically, technologies that can detect a greater breadth of substances and quantify the amount of substance present. Major technologies of interest included FTIR and MS. When asked what an ideal drug checking service looks like, all SSP staff members mentioned the acquisition of FTIR technologies. Recognized benefits include the non-destructive nature of the testing, lower acquisition cost and cost per sample processed, and perceived trust in the results. While MS destroys the sample during testing, FTIR does not, which could allow for the return of the sample to the client depending upon the legality of doing so. One participant noted how this non-destructive nature could be useful for training, allowing one sample to be repeatedly tested.

Participants noted limitations to the technologies discussed, including technical limitations that require confirmatory lab-based testing, which will be discussed further in the section *Confirmatory Testing*. Another important consideration from an experienced drug checker was the availability of validation data for technologies of interest and concern over the quality of new machines entering the market.

“We are seeing a lot of start-up companies pushing unvalidated machines. People are very aware that there's a lot of money coming into the sector right now. So everyone is trying to posture themselves to collect that money and not all of the machines that are being pushed are worth their cost.”—Participant #9

#### Confirmatory testing

Confirmatory testing refers to the use of gold-standard laboratory-based methods for identification. Participants utilized confirmatory testing through partnerships with outside laboratories. However, utilization of sanctioned laboratories or technologies owned by other drug checking services may be cost-prohibitive and time-consuming. Participants also described partnerships with university laboratories, including unsanctioned use, and the personal risk they assume when transporting illegal substances (such as felony charges for mailing substances). This apprehension was also felt by clients when sending their samples to other locations. To combat some existing barriers to confirmatory testing, participants suggested partnerships with in-state institutions or a centralized testing network where various SSPs could send samples for confirmatory testing and receive results in a timely manner.

The importance of confirmatory testing was described by participants in two major ways: for confirmation of samples and technician training purposes. Confirmatory testing for samples aids in the identification of confusing samples, or those which exceed the specifications of the testing method used (e.g., FTIR limit of detection). When training drug checking technicians, confirmatory testing can provide the “truth,” and ensure technicians are trained properly.

“We can see the concordance between our FTIR results and GC-MS. If there are organizations that aren’t doing that, they will never actually know if they’re right or wrong...”—Participant #9

#### Location

Participants discussed the formation of “nodes” when determining the most appropriate locations for drug checking technologies within the state. “Nodes” refer to locations that would maximize the public health impact of drug checking and be preferred candidates for resource allocation (i.e., drug checking technologies, funding). Participants frequently described obtaining geographic coverage through strategic placement of “nodes” to ensure adequate access for major populated areas and to facilitate identification and tracking of drug trends (discussed further in Data Sharing section).

When determining locations for drug checking services, the first major consideration is the potential impact of placement in an area. The second major consideration is the existence of an established harm reduction organization that could effectively provide drug checking services. Utilization of existing services was thought to be important because these agencies have already established trust and comfort among clients, which is important for client engagement and may lead to faster uptake of new drug checking services. When discussing processes for the selection of candidate organizations for drug checking, caution was expressed in letting organizations self-determine their capability, citing a lack of understanding of the complexities involved, and a lack of capacity for execution.

“Harm reduction is more complex than a lot of organizations think. We get inundated with requests a lot just to do harm reduction work… and they just don't have the capacity, at least for our standards, to do it.”—Participant #8

#### Funding

Funding for harm reduction services was seen as an important facilitator. Participants reported a general lack of funding for services and the historical reliance on HIV grant funding for survival. Participants also described legal limitations related to grant funding and the inability to use funds for many harm reduction purposes because the supplies are federally illegal. Consideration of funding sources for drug checking is essential to ensure adequate support for programs. Similar considerations may also be relevant to partnerships, such as universities, which may receive federal funding that restricts their ability to provide confirmatory testing services.

When discussing funding related to drug checking specifically, participants noted that capital costs are not the predominant source of the investment; rather, it is the staff necessary to operate the technologies and provide the service. Participants emphasized that support should not only encompass the initial investment in the drug checking technology but also include training and compensation for staff, reference libraries, support costs for the technology over time, and vehicles equipped to offer mobile drug checking.

It is also important to consider the infrastructure of existing harm reduction organizations and recognize the variation between agencies. This has important implications for individualizing support for agencies with different funding needs. Reliable funding is critical to program sustainability, which participants described as essential to ensuring clients receive continued support, and prevent the disenfranchisement of clients who relied upon services only for them to be terminated.

“This crucial lifeline that we relied upon, would just disappear and dry up and then that further disenfranchises people from wanting to engage with services.”—Participant #7

#### Integration of services

Participants highlighted how integrating drug checking with other services can provide two major benefits. First, offering a variety of services can provide plausible deniability, the concept that a client could be at a location for several reasons other than drug checking. Participants identified this as important, given barriers for clients related to stigma and surveillance. Additionally, integrating services can help staff better serve their clients by meeting needs beyond drug checking.

“Part of the purpose of harm reduction is to try to get people plugged into services that are not actively receiving services… So if we integrate drug checking… they are more likely to take home the Naloxone, they're more likely to see a psychiatrist, they're more likely to receive primary healthcare, and all of these components lead to an individual being healthier.”—Participant #7

Participants described how integration could help to ensure that clients receive services, noting that “if we're sending them somewhere else to have the services done, they may not make it or they may not go.”—Participant #3.

#### Staff: experience and training

Incorporating staff members who are familiar with the community may help facilitate a quicker and more successful implementation of drug checking services. Participants described how lived experience creates an existing foundation of knowledge and helps to establish trust and rapport with clients, leading to smoother transitions. Drug checking technicians with lived experience were also seen as more likely to be retained assets. Concerns were expressed for long-term retention of drug checking technicians from more traditional academic or research backgrounds, who may be more likely to view the position as a stepping stone in their career path. This was felt to be detrimental to the drug checking services, which could be left with a significant resource and knowledge void.

“When you have researchers or other academic technicians come into the space… you have the risk of them then leaving for a different kind of research job and taking all of those resources and knowledge with them. So then you leave the harm reduction community with this void, and that's hazardous. Training people in the community is beneficial for the community and it keeps it running.”—Participant #9

However, care should be exercised when selecting drug checking technicians. Participants recognized that drug checking is a technically challenging position and requires someone capable of acquiring the expertise necessary. One participant already had a candidate in mind for a future drug checking service, while another described how they used experience with organizational affiliates to help identify and select interested and capable people.

Concern was expressed over inadequately trained technicians providing inaccurate results to clients, with serious health implications. The utilization of confirmatory testing was identified as an important facet of technician training to ensure that the interpretation of results is done correctly and that clients receive accurate information. Participants also emphasized the desire for standardization within the field and the lack of best practices for drug checking technician selection and training.

“I really hope this happens eventually, but there is some sort of accreditation process towards being a drug checker… if you don't know how to accurately interpret the data, you could be providing something that's really dangerous to your participants.”—Participant #9

### Macro-level themes

#### Data sharing

Strong interest was expressed in sharing data obtained from drug checking services. Participants highlighted how the positive impact of drug checking could extend far beyond the individual client utilizing the drug checking service. One participant described how engaging and performing drug checking for distributors can impact many people who use that supply and how the data obtained from services can benefit communities across the data sharing network.

“On one hand, I could look at it and say we have 47 unique participants that have utilized our service... But I can look on the other end of it and recognize that 4 of those participants are distributors whose supply touches as many people. Then I can look at 3rd party data coming from other agencies that benefits that entire network and it gives them a better understanding. So I could stay 47, or I could say thousands.”—Participant #9

The creation of a statewide database was recommended to facilitate information sharing among harm reduction organizations. This information was described as critical for tracking drug trends across the state and allowing organizations to inform and serve their clients properly. Challenges with collecting and managing data were recognized. For data sharing to be successful, the database should be managed by an experienced and capable partner. As one participant noted “Us managing the data system on our own would be challenging I think, and so having… it managed by an actual source that really knows what they're doing, I think would be a great thing to have.”—Participant #6. Furthermore, standardization across organizations should be implemented to ensure that data is comparable and useful.

Participants suggested several variables that would be important for data collection. These include what the substance was sold as, its appearance, location of purchase or testing, testing results (substances, quantities, etc.), and effects experienced (if the substance was previously used). Preserving clients' anonymity was seen as critical, and participants strongly discouraged the collection of identifiable information. Consideration was also given to data access. Harm reduction organizations were seen as entities that could benefit from access and facilitate information sharing within their communities (e.g., “bad batches,” drug trends). Strong concerns were expressed over the potential misuse of the data by law enforcement, who may use it for tactical purposes, which could erode trust from clients.

#### Partnerships

During the interviews, discussions on the utility of partnerships between harm reduction organizations and other entities arose many times. Key areas where partnerships may be impactful are in confirmatory testing (utilization of existing confirmation services, partnerships with university laboratories, or equipping an SSP to provide centralized services) and data sharing (creation of a statewide database), as noted above. In addition, participants described how partnerships between harm reduction organizations could help facilitate drug checking outreach in more rural areas:

“You'd have to develop some kind of infrastructure if someone is in an outlying county that doesn't have access to your device right away. How do we get that to them? How do they get the samples to us? And how do we get their information back to them in a very timely manner? That can be worked out through partnerships.” - Participant #2

Participants also described how partnerships could help facilitate inter-organization communication to foster more successful programs and assist newer programs by sharing procedural data and lessons learned.

#### Legality

Fear of criminalization and prosecution were described as primary barriers for clients utilizing drug checking services. Participants emphasized existing disbelief from clients regarding the legal status of drug checking and existing legal protections in place (decriminalization). Moreover, participants described the historically tense relationship between law enforcement and PWUD, a lack of sympathy from law enforcement, and how arrests for possession of substances for drug checking continue, despite decriminalization bills.

“I think getting people to trust this is going to be a challenge...And even though we have state bills that prohibit prosecution from residual, by definition, residual amounts of drug possession for checking purposes we know people are still getting arrested for it.”—Participant #7

The legal risks associated with drug checking also extend to the staff of drug checking services, who assume significant risk to provide services to clients. This risk is mainly associated with transporting substances for confirmatory testing purposes, which has major implications for creating a more centralized testing network or partnerships with in-state institutions.

#### Legitimacy

Participants identified the need to promote the legitimacy of drug checking services through public support from state agencies. Criminalization concerns remain a major barrier to service utilization by clients (described above). Despite decriminalization, participants reported disbelief from their communities, citing a lack of promotion or support as a key barrier. Participants described how support from state agencies, like public health departments, can build trust and awareness, such as with the promotion of other harm reduction services like testing for sexually transmitted infections.

“When you have large corporations like [large healthcare systems], health care centers, or the [local] public health department, come out in support of us, that carries a lot of weight here locally.”—Participant #2

However, a fine line exists between advocacy and direct agency involvement which could be detrimental to trust from participants. As one participant noted, if “the government is coming with us, people are going to be like, we don't trust you.”—Participant #8.

#### Advisory panel

The network of harm reduction organizations and employees across the state was described as collaborative. Participants explained how they must rely on one another for information sharing because formal resources and information for drug checking are not readily available, noting that *“if we waited for resources and knowledge and toolkits and all of that stuff, we'd still be waiting.”*—Participant #1. Several SSP staff members mentioned the Alliance for Collaborative Drug Checking (ACDC), an international coalition for drug checkers that facilitates information sharing on drug checking technologies, trends, and experiences.

Creating a formal advisory panel may promote successful program implementation. Understanding the capability and capacity of harm reduction organizations and the local need is crucial. Utilizing local, boots-on-the-ground harm reduction staff may be a beneficial resource. When discussing the formation of a state advisory panel, participants cautioned against the inclusion of members who may have conflicts of interest, such as organizations that profit from supplies used for drug checking or those who lack sensitivity to the opioid epidemic.

“I work as a contractor with them [organization]. But they're very much focused on the recreational side and the psychedelic side. So if they came in with any policy kind of “woo woo” stuff I feel like it would currently be a distraction. I'm not saying that they're a bad organization in any regard. I just think that we need to have a focus on the opioid supply… There's some groups that are just heavily research focused and centered and they get excited when something like xylazine comes into the scene and they get excited because it's a study opportunity and it's pardon my language, but it's [expletive redacted].”—Participant #9.

## Discussion

The purpose of this evaluation was to inform decision making on policy and funding for drug checking services, which followed a multi-year pilot of drug checking in an urban harm reduction agency. Key aspects of drug checking services that agencies, such as state health departments, can support included utilization of advanced mobile testing, confirmatory testing, sustained funding for technology and staff, strategies to improve utilization, and raising public awareness of the legitimacy of harm reduction strategies, including drug checking services.

This study contributes a US perspective to a literature base of predominantly European-based studies [13]. As the opioid epidemic continues to persist and evolve across the USA, the finding presented here may help frame considerations important to similar populations and drug markets. However, many of the identified themes, particularly those at the client or drug checking service level (e.g., accessibility, privacy, integration of services), may be more broadly generalizable.

We conducted key informant interviews with stakeholders experienced in harm reduction and drug checking services; many offered a unique perspective based on lived experience. Conversations facilitated the identification and discussion of many contextual factors critical to drug checking services. This resulted in a more holistic evaluation and expanded on the typical health technology assessment focused on the costs and benefits of a given technology.

Many important considerations were identified by participants across the spectrum of micro-, meso-, and macro-level themes. FTIR was described as a desirable drug checking technology owing to its quantitative capability, breadth of detection, portability, and non-destructive nature; considerations supported by findings from prior studies [8, 14, 15, 25]. Participants described comfortability with FTIR and the existence of validation data for its use in harm reduction settings. This suggests the need for future replication as experience with technologies continues to evolve, and as new technologies enter the marketplace.

Confirmatory testing has been described as a method to overcome the technical limitations of drug checking technologies [26, 27]. Participants also stressed the need for standardized technician training programs, which may be improved with confirmatory testing as a “check” on the technician’s work. The British Columbia Centre on Substance Use requires drug checking technicians complete modules through an online learning platform and have 30 shadowing hours with a sponsoring organization [28]. Such programs could provide a source for training emulation. Participants identified several methods for confirmatory testing, including the use of existing sanctioned laboratories or through partnerships with local resources such as universities or existing drug checking services. The most appropriate method of confirmatory testing could vary based on the anticipated volume of samples, budget, training needs, and presence of local resources, and should be considered on an individualized basis.

Many important considerations were discussed by participants regarding drug checking service utilization. Stigma and criminalization continue to be major barriers, as widely described in the literature [8, 18, 29–31]. Legitimization through public displays of support by the state was described as an important opportunity for improvement. Given the variable legal status of drug checking technologies across the USA [32], risks to staff members and clients must be considered within the context of the local jurisdiction. This has important policy implications on confirmatory testing and whether drug checking services have legal protections to transport substances for testing or if additional protections are needed.

Other important considerations include service location, establishment of trust with clients, and sustainability of services. Locations should leverage existing harm reduction agencies and be in an area that maximizes the potential public health impact while balancing access for rural communities. This highlights the importance of conducting a local needs assessment to understand and identify impactful locations. Staff members with lived experience were seen as critical to establishing rapport and trust with clients. People with lived experience were also seen as dedicated and able to offer many important qualities as drug checking technicians. However, questions remain on how to identify and select drug checking technicians. Service sustainability is not only crucial to ensure clients receive continued support, but to prevent the disenfranchisement of clients who relied upon services only for them to be terminated. Funding is one of the prominent factors when considering long-term sustainability of a harm reduction service. The participants engaged in this study had variable staff sizes, resources, and infrastructure for drug checking, highlighting the need for individualized support when implementing new drug checking services.

Consultation with existing harm reduction organizations is a critical step in needs assessment, informing resource allocation, and developing a strategy for implementing drug checking technologies. The formation of an advisory panel for drug checking could be an important resource in the implementation process. Ideally, this should incorporate local harm reduction agencies with an in-depth understanding of the local community and existing barriers.

### Limitations

In identifying key considerations for the implementation of drug checking services, we would note that only one drug checking service had direct experience with quantitative technologies. However, all participants from drug checking services were well versed in the available technologies and were actively pursuing funding for technology acquisition. Another limitation of note was that participants were located in the US Midwest, specifically in Illinois. However, participants were located in major urban as well as rural settings which may support the generalizability of themes and considerations, which may apply more broadly to regions across the USA.

## Conclusions

Decision making around drug checking service implementation involves considerations beyond a given technology's acquisition cost and specifications. It also requires knowledge of local needs and capacity, and an in-depth understanding of the target population. Key informant interviews helped to identify important criteria in valuing and adopting a drug checking technology (e.g., accuracy, breadth of compounds, acquisition and maintenance costs, technical knowledge, ability to hire and retain technical staff) that may overlap with criteria of the agencies that provide those services (e.g., accessibility, privacy, trustworthiness, legitimacy, sustainability). Consultation with leadership at harm reduction organizations can provide tremendous insight into the principles underlying drug checking services and raise pragmatic considerations for program implementation.

## Data Availability

The datasets used and/or analyzed during the current study are available from the corresponding author on reasonable request.

## Abbreviations

ACDC: Alliance for Collaborative Drug Checking
CDC: Centers for Disease Control and Prevention
CFIR: Consolidated Framework for Implementation Research
FTIR: Fourier transform infrared spectroscopy
GC–MS: Gas chromatography with mass spectrometry
IDPH: Illinois Department of Public Health
MS: Mass spectrometry
OD2A: Overdose Data to Action
PWUD: People who use drugs
PWUPD: People who use party drugs
SSP: Syringe service program
